# Adaptive Strategies Mediating the Diversification of Alpine Plants: The Case of the Himalayan Blue Poppy (*Meconopsis*, Papaveraceae)

**DOI:** 10.3390/plants14243741

**Published:** 2025-12-08

**Authors:** Na He, Zhimin Li, Yazhou Zhang, Wenguang Sun

**Affiliations:** 1 School of Life Sciences, Yunnan Normal University, Kunming 650500, China; hena_luc@163.com (N.H.); lizhimin_vip@163.com (Z.L.); 2 Yunnan Key Laboratory of Plant Diversity and Biogeography of East Asia, Kunming Institute of Botany, Chinese Academy of Sciences, Kunming 650201, China

**Keywords:** trait evolution, species diversification, *Meconopsis*, reproductive traits, ploidy, Qinghai-Xizang Plateau

## Abstract

Alpine habitats, characterized by their high degree of environmental heterogeneity and harsh climatic conditions, support a diverse array of plants with unique adaptive strategies. However, the mechanisms underlying the formation of these adaptive strategies, as well as their intrinsic links to species diversification, remain unclear. In this study, we investigated the evolution of life history traits, fruit characteristics, and variation in the karyotype of alpine species, and their roles in shaping their adaptability to high-altitude environments. We performed a comprehensive analysis of trait diversification, adaptive trait evolution, and their associations with environmental factors in the genus *Meconopsis* on the Qinghai-Xizang Plateau. Our results revealed that ancestral floral traits were characterized by solitary inflorescences and blue-purple pigmentation, features that have re-evolved at multiple points throughout the evolutionary history of the genus. We found that increased ploidy levels promoted perennial growth and semelparity (single-time fruiting), suggesting that life history strategies and fruiting frequency are strongly coupled. Furthermore, karyotypic variation and abiotic factors such as altitude, soil pH, and climate were found to accelerate the evolution of a perennial fruiting reproductive strategy. Our findings provide new insights into the evolution of adaptive traits in alpine plants and reveal how these species adjust their life history strategies in response to environmental pressures. Our findings enhance our understanding of resource allocation trade-offs in plants in extreme environments and shed light on the relationship between species diversification and adaptive evolution in alpine ecosystems.

## 1. Introduction

Alpine plants, inhabiting environments characterized by extreme climatic conditions and pronounced environmental heterogeneity, exhibit diverse adaptive strategies to cope with these challenges [1]. However, the evolutionary mechanisms driving the formation of such strategies and their connections to species diversification remain insufficiently understood. In this study, we investigate the evolution of life history traits, fruit characteristics, and karyotypic variation in alpine species of the genus *Meconopsis*, assessing their roles in adaptation to high-altitude environments.

*Meconopsis*, commonly known as the Himalayan blue poppy, is renowned for its striking floral displays and horticultural importance. According to the Flora of China, the genus comprises approximately 54 species, predominantly distributed in the Sino-Himalayan region, with a single species found in Western Europe. China alone harbors 43 species thriving in subnival and alpine meadow habitats above 3000 m, where plants endure low atmospheric pressure, intense ultraviolet radiation, prolonged sunlight, and large diurnal temperature fluctuations [2]. The genus has attracted increasing research interest recently [3], not only for its ornamental value but also for its rich species diversity and complex reproductive strategies, making it an excellent model for studying environmental adaptation, ecological niche dynamics, genetic diversity, and ecosystem stability in alpine regions.

Despite extensive taxonomic documentation, the evolutionary trajectories of *Meconopsis* morphological traits remain complex and partially unresolved. In particular, the relationship between floral evolution—such as color and inflorescence structure—and geographic expansion warrants further investigation. Polyploidy is a prevalent evolutionary mechanism in the Qinghai–Tibetan Plateau (QTP) flora, frequently driving speciation and ecological adaptation. Yet, the interplay between ploidy levels and environmental factors influencing vegetative growth and reproductive success in *Meconopsis* remains unclear. To address these gaps, we hypothesize that while the center of origin for *Meconopsis* may be ancient, the subsequent environmental dynamism resulting from orogeny significantly promoted chromosomal diversity and speciation. Furthermore, blue-purple flowers, solitary inflorescences, and an annual/biennial life history are the key evolutionary innovations fueling the genus’s rapid radiation.

The diversity of alpine plants and their adaptive evolutionary mechanisms have long been central topics in ecology and evolutionary biology [4,5]. The Qinghai-Xizang Plateau (QXP), the highest and largest plateau in the world, often referred to as the “Roof of the World,” harbors ecosystems and plant communities of exceptional scientific value due to its unique geographic and climatic conditions [6]. Within this region, the Hengduan Mountains (HDM) are a particularly significant area with numerous “sky islands.” These “sky islands” are isolated high-elevation peaks with distinct geographic features that create diverse and heterogeneous habitats. Spatial isolation and frequent climatic fluctuations in these areas promote repeated cycles of population mixing, isolation, and remixing, which are known as Mixing-Isolation-Mixing (MIM) cycles [7]. This process drives species differentiation and fosters biodiversity, making such peaks invaluable natural laboratories for biodiversity research. However, the geographic distribution patterns of alpine plants on the QXP, as well as the effects of interactions between adaptive traits (e.g., life history, floral characteristics, and karyotype) and abiotic factors (e.g., climate and soil properties) on resource allocation, especially trade-offs between growth and reproduction, remain poorly understood.

Life history differentiation is one of the key adaptive strategies employed by alpine plants in response to heterogeneous habitats [8]. For example, perennial herbs mitigate constraints on the growing season by storing carbon in underground organs (e.g., tubers or rhizomes), which facilitates inter-annual resource allocation [9]. Recent findings highlight a strong correlation between life history shifts and altitude, wherein the dominant plant species shift from woody shrubs to perennial herbs as elevation increases [10]. In addition, the reproductive organs of alpine plants exhibit substantial morphological variation and are ecologically specialized, which reflects their adaptive importance. Floral traits serve complex functions to attract specific pollinators and ensure effective pollen transfer [11,12], which is especially critical for alpine species. Reproductive traits in these environments show strong patterns of evolutionary convergence; for example, 67% of species bear blue-purple flowers with ultraviolet reflectance exceeding 80%, which enhances pollinator visual recognition and pollination efficiency [13]. Solitary flower sequences also increase with altitude, potentially reducing damage to reproductive tissues from frost [14]. Furthermore, cleistogamy (self-fertilization within closed flowers) occurs 3.8 times more frequently in extreme habitats than in habitats without extreme conditions, suggesting that there is a trade-off between reproductive security and genetic diversity [15].

Polyploidy plays a significant role in the genomic evolution of alpine plants and occurs 2.3 times more frequently in alpine species than in lowland species [16]. Whole-genome duplication contributes to genetic redundancy and enhances evolutionary flexibility through several mechanisms: (1) it increases plasticity in the expression of cold-response genes (e.g., CBF transcription factors) [17]; (2) it strengthens metabolic buffering, such as in flavonoid biosynthesis pathways [18]; and (3) it facilitates subgenome divergence, accelerating the fixation of adaptive alleles [19]. Chromosomal polyploidy, a key evolutionary mechanism, leads to extensive morphological, physiological, biochemical, and adaptive changes [20]. Variation in the number of chromosomes can promote radial speciation [21], and polyploidy is widely observed across green plant lineages [22,23], where it is recognized as a major driver of evolutionary change [24,25]. Changes in chromosome motifs can affect genome size and structure and influence plant morphology, biomass (e.g., fruit size), and geographic distributions [26,27]. Numerous studies have revealed strong associations of plant diversity with variation in chromosome base numbers and ploidy levels [28,29]. Although the importance of polyploidy in plant diversification is well established [30], its specific effect on reproductive and nutritional traits in alpine plants remains poorly understood.

In this study, we constructed phylogenetic trees based on chloroplast marker genes, calibrated divergence times using fossil records, and reconstructed the ancestral geographic range of *Meconopsis*. We also mapped key reproductive traits, including inflorescence structure, flower color, and life history types (annual vs. perennial; single vs. multiple fruiting), tracing the shift in reproductive traits from ancestral to derived forms. This integrative approach allowed us to explore the evolution of reproductive traits, growth patterns, and fruiting strategies of *Meconopsis* lineages, as well as their geographic distributions. Additionally, we compiled species distribution data and extracted 43 environmental variables to analyze multi-dimensional patterns of growth and fruiting across different clades and karyotypes. Specifically, we aimed to (1) investigate the regional differences in adaptive outcomes and diversification rates among *Meconopsis* lineages across the Qinghai–Tibet Plateau (QTP); (2) determine the primary intrinsic and extrinsic influences driving the adaptive evolution of reproductive traits in *Meconopsis*; (3) identify which reproductive trait states (e.g., flower color, life history) serve as key evolutionary drivers of speciation and diversification within the genus *Meconopsis*. Our findings provide valuable insights into the trade-offs between growth and reproduction in alpine plants and enhance our understanding of the adaptive strategies employed by alpine plants in response to environmental stress.

## 2. Results

### 2.1. Ancestral Distribution Regions and Divergence Time Estimation

**Figure 1:**
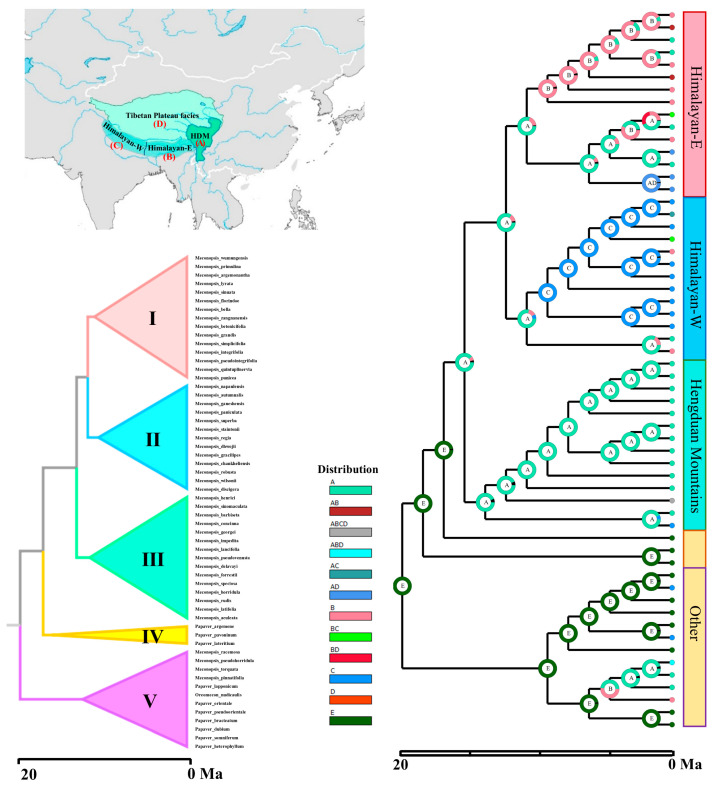
Phylogenetic chronology and ancestral range estimation of *Meconopsis* plants. A maximum likelihood phylogenetic tree constructed based on the *Meconopsis* chloroplast gene sequences was used to generate the timing maps using BEAST, as shown in Supplementary Data Figures S1–S3. The map on the top left shows the approximate delineation of the species region, and the tree on the right shows the topology of the ML tree, with different colors representing different branches. Ancestral range of *Meconopsis* estimated using RASP under the BAYAREALIKE+J model, maxarea = 2 is represented by colored circles at each node, numbers in the figure represent millions of years, Roman numerals for clades represent different branches, and the text on the far right is labeled according to the major geographic locations in the *Meconopsis* clade. Regions are defined as follows: A, HDM; B, Eastern Himalayas; C, Western Himalayas; D, the platform of the Qinghai-Tibet Plateau; and E, Asian regions excluding the QTP. Roman numerals I–V represent the five major lineages, with V primarily representing the outgroup.

We first constructed ML and BI phylogenetic trees with a high overall resolution (Figures S1 and S2). The results showed that approximately 90.24% of the nodes received support values of ≥75%, with most nodes exhibiting high support and confidence levels (Figure 1). Phylogenetic analysis revealed that *Meconopsis* and its closely related genera were divided into five major clades. Temporal divergence estimates based on the plastid dataset indicated that the origin of *Meconopsis* occurred approximately 20.6 million years ago (Ma), with a 95% HPD interval closely aligning with previous findings [31]. Model selection results for the ancestral region reconstruction are shown in Table S1. Species in Clade I were primarily concentrated in the eastern Himalayas (Himalayan-E), those in Clade II were primarily concentrated in the western Himalayas (Himalayan-W), and those in Clade III were primarily concentrated in the HDM (HDM). Species in Clades IV and V were distributed in non-identified areas (Other), with *Meconopsis* primarily distributed across the QXP facies and the HDM. These five branches are also shown on the map (Figure 1), with further details provided in Table S2. Notably, Clades II and III received 100% support, which indicates high confidence in their topological position.

### 2.2. Evolution of Reproductive Traits

**Figure 2:**
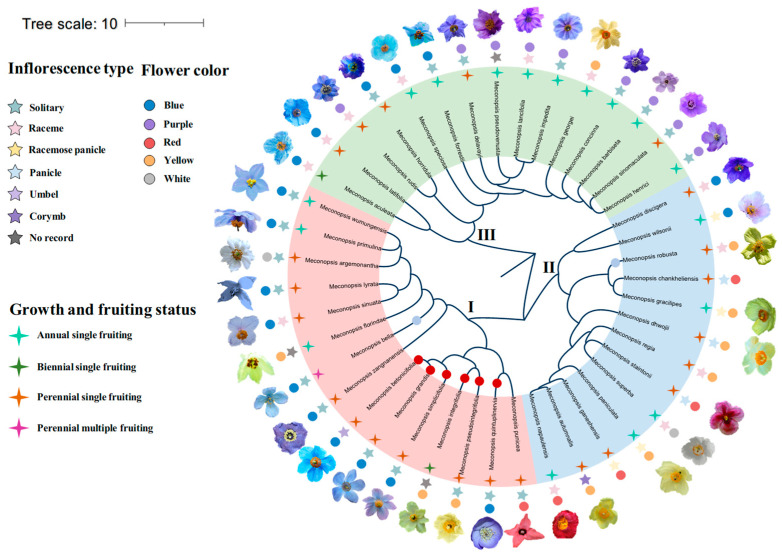
Mapping of *Meconopsis* inflorescence, flower color ancestry and growth and fruiting. The phylogenetic tree constructed based on the great likelihood method, with feature mapping of three key characteristics of the three main branches of *Meconopsis*, shows the most probable ancestral traits and the evolutionary patterns of different characteristics in different clades. Pentagrams represent inflorescence type, circles represent flower color, stars represent growth and fruiting, different colors represent different states, and colored circles and Roman characters represent different clades. The solid circles in the middle of the branches represent the changes in chromosome numbers recorded so far. Red indicates an increase in chromosome numbers, while blue indicates a decrease in chromosome numbers. Most of the unmarked chromosomes are 56 in number (including those that have not been recorded).

Character mapping of inflorescence types across the three clades of the genus *Meconopsis* (Figure 2) indicated that the ancestral state was most likely a unifloral inflorescence. All inflorescence types found in the basal taxa of *Meconopsis* are unifloral, representing approximately 42.55% of the taxa sampled within the group. Independent transitions from solitary to racemose inflorescences occurred in Clade I, while transitions from racemose back to solitary inflorescences occurred in Clade III at least twice during their evolutionary history. Mapping of floral color traits revealed that the ancestral state was most likely blue-purple, a trait observed in 63.83% of the sampled *Meconopsis* taxa. In Clade I, blue flowers evolved into yellow or red flowers on at least three separate occasions. Annual species with one-time fruiting were predominantly concentrated in Clade III, whereas perennial one-time fruiting species were primarily found in Clades I and II. Within Clade I, perennial one-time fruiting underwent at least two transitions to annual one-time fruiting; in Clade II, at least three such transitions occurred. In contrast, Clade III exhibited transitions from annual to perennial growth.

### 2.3. Effects of Abiotic Factors on Reproductive Traits

**Figure 3:**
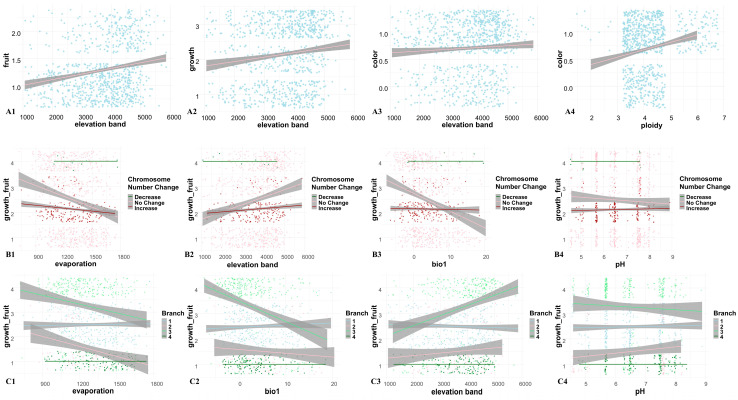
Mixed effects model to analyze the trend of different species of *Meconopsis* under different environments and ploidy changes. The horizontal coordinates represent the independent variables and the vertical coordinates represent the dependent variables. (**A1**–**A4**) selected elevation, ploidy, color, growth years, and number of fruiting for linear regression from the relationship plot between variables (Figure S4). (**B1**–**B4**) classified the changes in chromosome base number into three categories (−1: decrease, 0: no change, 1: increase) and analyzed the trends of growth and fruiting in different environmental factors using mixed linear models. (**C1**–**C4**) classify the four major clades (1: clade I, 2: clade II, 3: clade III, 4: clade IV), and analyze the trend of growth and nodulation in different environmental factors using mixed linear models. The points of different colors represent the status of *Meconopsis* under different clades, and the direction of the lines represents the change trend. The specific meanings of horizontal and vertical coordinates are shown in Table S3.

**Figure 4:**
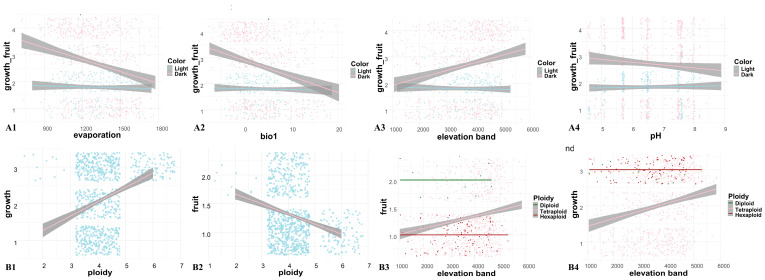
Mixed effects model to analyze the trend of different species of *Meconopsis* under different environment and ploidy changes. The horizontal coordinates represent the independent variables and the vertical coordinates represent the dependent variables. (**A1**–**A4**) classify the flower color shade (1: dark, 0: light) and analyze the trend of growth and fruiting in different environmental factors using mixed linear models. (**B1**,**B2**) correlation analysis of ploidy with growth years and number of fruiting, (**B1**) horizontal coordinate is chromosome ploidy, vertical coordinate is growth years, vertical coordinate of (**B2**) is number of fruiting, and the line represents the trend of change. (**B3**,**B4**) classify chromosome ploidy into three categories (2: diploid, 4: tetraploid, and 6: hexaploid), and analyze the trends of growth and fruiting in elevation using mixed linear models.

A heatmap was first generated (Figure S4), which revealed 10 variables with high correlations at the genus level. These data were then visualized to make preliminary observations (Figure S5), which highlighted interrelationships among the variables. To further explore these associations, we separately plotted the relationships between abiotic factors, trait characteristics, and karyotypic traits (Figure 3, Figure 4, Figures S5 and S6). While slight variation in trends was observed across different clades or ploidy levels, these did not affect the overall pattern. The results revealed that increases in elevation and chromosome polyploidy have driven the evolution of perennial fruiting in *Meconopsis* species, and in some cases, toward the evolution of multiple fruiting events. When environmental conditions such as elevated potential evapotranspiration, higher mean annual temperature, and higher soil pH are interpreted as adverse, these stressors appear to promote the evolution of annual species with single-time fruiting.

To assess whether chromosomal diversity, growth and fruiting are spatially correlated, spatial autocorrelation analyses were conducted using the spdep package by comparing three models: OLS, SLMs, and SEMs (Table S4). The results demonstrated clear spatial autocorrelation in the influence of karyotypic diversity, specifically chromosome number, on growth and fruiting. Chromosome number was projected onto a geographic map (Figure S8), which revealed significant chromosomal diversity in *Meconopsis* species within a distinct geographic region at the junction of the eastern and western Himalayas, particularly around Mount Qomolangma.

### 2.4. Multifactor Correlation Analysis

The 49 ecological variables were grouped into four categories, biological, climatic, soil, and terrain, for dimensionality reduction analysis. The cumulative variance explained by the PCA and the goodness-of-fit assessment are illustrated (Figure S9). As shown in the results (Figure S10), sister taxa from different *Meconopsis* clades exhibited varying responses to the different types of ecological factors. Clades III and IV (groups C and D) showed relatively minor differences in climate, soil, and topography, followed by clade I (group A), and clade II (group B) displayed the most pronounced differences. Moreover, variation in biological factors, such as phenotypic traits and karyotypes, was also evident.

Further analysis revealed that species with different growth and fruiting conditions differed in reproductive traits and karyotypes (biological factors), whereas species with similar growth and fruiting characteristics tended to occupy comparable ecological niches. Notably, clades III and IV (groups C and D) demonstrated a high degree of ecological similarity.

### 2.5. High Speciation Rates Are Associated with High Net Diversification Rates

**Figure 5:**
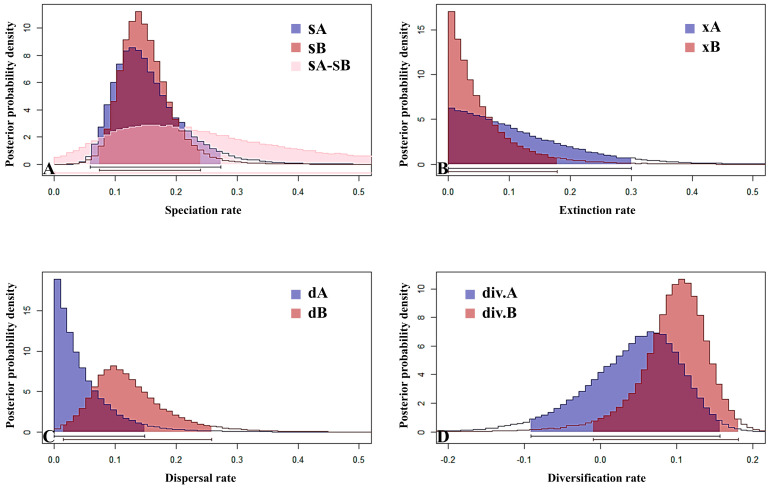
Geographical distribution and diversification. The horizontal axis represents the rate, while the vertical axis represents the posterior probability. The vertical axis in all the figures represents posterior probability density. In (**A**), the horizontal axis represents the speciation rate: sA is the speciation rate in area A (HDM), sB is the speciation rate in other areas (mainly the Himalayas), and sA-sB is the shared speciation rate between areas A and B. In (**B**), the horizontal axis represents the extinction rate: xA is the extinction rate in area A, and xB is the extinction rate in area B. In (**C**), the horizontal axis represents the dispersal rate: dA is the dispersal rate in area A, and dB is the dispersal rate in area B. In (**D**), the horizontal axis represents the diversification net: div.A refers to the diversification rate in area A, and div.B refers to the diversification rate in area B. The line segments at the bottom of the figures represent the range of the density functions. In Figure (**A**), the distributions of SA and SB exhibit substantial overlap, indicating that the speciation rates of Clades A and B show statistically minor differences. In contrast, in Figure (**C**), the distributions of dA (blue) and dB (brown) display limited overlap, which suggests a significant difference in dispersal rates between these two clades.

We employed a diversification rate model to quantitatively estimate the speciation, extinction, and dispersal rates of *Meconopsis* species in the HDM region (area A) and the Himalayan-dominated region (area B) (Figure 5). Diversification rates were also estimated for floral color and inflorescence traits. The results revealed differences in species diversity and evolutionary dynamics between the two regions. Specifically, area A exhibited relatively lower speciation, dispersal, and overall diversification rates compared with area B, while extinction rates were similar between the two. These patterns may be closely linked to climatic fluctuations since the late Cenozoic, such as the glacial–interglacial cycles of the Quaternary. The lack of stability associated with these climatic shifts may have led to frequent extinction events and potentially accelerated speciation through the intensification of natural selection. Although the HDM are topographically complex, they have likely acted as a relatively stable refugium. In contrast, the Himalayas may have undergone more intense recent geological uplift and climatic dynamism. This severe environmental instability often drives accelerated speciation because populations are frequently isolated and subjected to secondary contact. The high speciation rate observed in the Himalayan region could further be promoted by factors such as a higher frequency of polyploidization events around Mount Everest or a greater degree of habitat fragmentation, both of which catalyze species formation.

**Figure 6:**
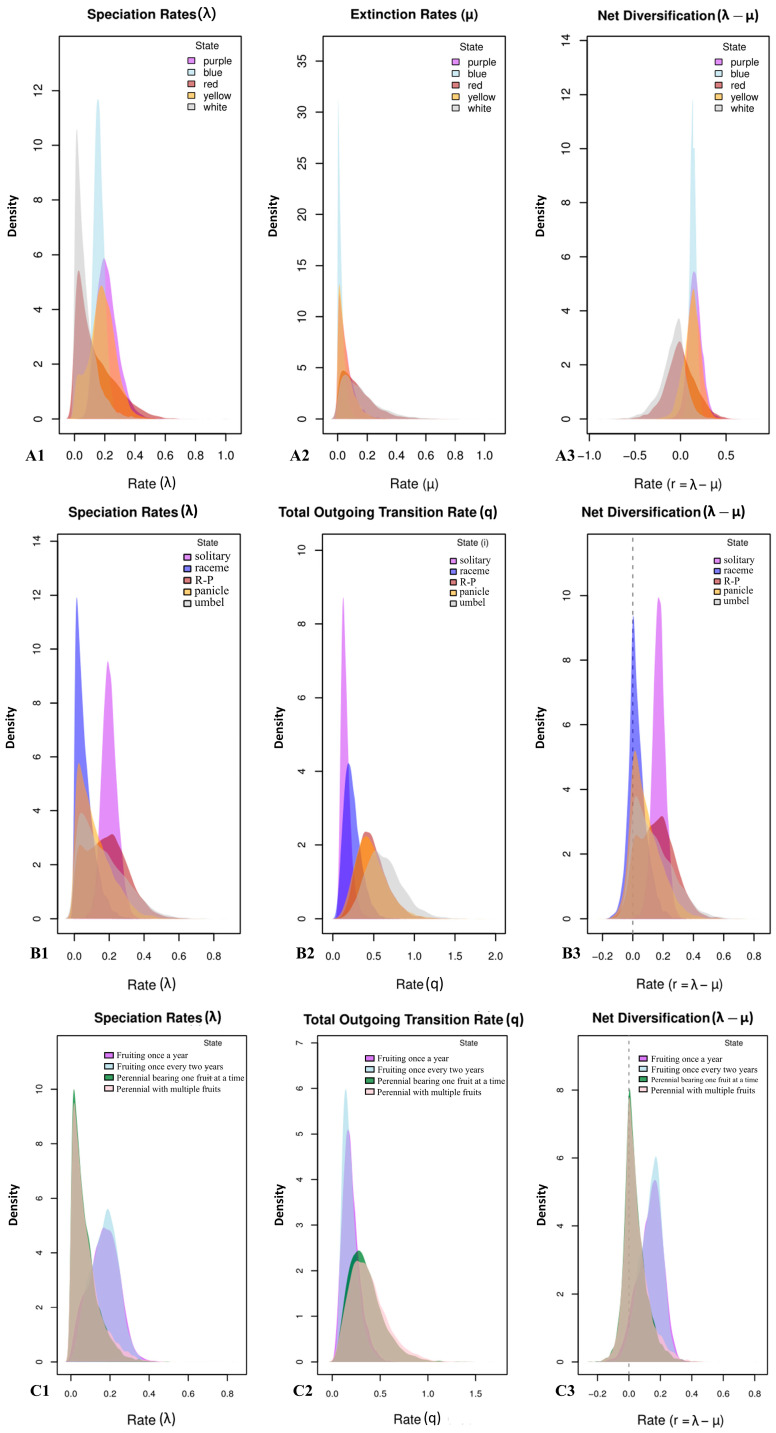
Diversification rates of reproductive traits. Horizontal coordinates represent rates and vertical coordinates represent probability densities, with higher peaks corresponding to more probable rates. (**A1**–**A3**) are the rates of species formation, extinction, and net diversification of species corresponding to flower color, respectively; (**B1**–**B3**) are the rates of species formation, dispersal, and net diversification of inflorescence types, respectively; and (**C1**–**C3**) are the rates of species formation, dispersal, and net diversification of growth and fruiting. The dashed line represents the position where the Net Diversification Rate is zero, indicating that the diversity is in a state of “dynamic equilibrium”.

Using the MuSSE model and posterior distributions from MCMC simulations (Figure 6), we further investigated the relationship between flower color, inflorescence type, growth form, fruiting strategy, and lineage diversification rates. Our findings indicate that lineages bearing purple, blue, or yellow flowers show similarly high species formation rates; solitary-flowered inflorescences are associated with the highest rates of speciation; and perennial primary fruiting exhibits the greatest speciation rate. Lineages with higher speciation rates generally also had higher net diversification rates within the *Meconopsis* taxa. These results suggest that floral color, inflorescence structure, and reproductive strategies may play important roles in shaping species formation dynamics in *Meconopsis*, leading to variation in species accumulation across lineages with different reproductive traits. The traits of blue-purple flowers, solitary inflorescences, and short-lived life cycles (annual/biennial) significantly promoted the diversification of the genus *Meconopsis*.

In high-altitude regions, pollinator abundance is low and the environment is harsh. The presence of blue-violet flowers in *Meconopsis* may more efficiently attract the limited available pollinators, thereby ensuring the formation and maintenance of reproductive isolation. Furthermore, anthocyanins may also play a role in UV radiation protection. In the extreme alpine scree environment, the growing season is exceptionally short and energy resources are severely limited. Plants tend to prioritize and concentrate major resources toward developing a single, large, frost-tolerant, and highly conspicuous flower. The short life cycle and rapid generation turnover of annual or biennial plants mean they accumulate genetic mutations faster and respond more rapidly to environmental selection pressures. This, in turn, accelerates the process of reproductive isolation and subsequent speciation.

## 3. Discussion

### 3.1. Geographic Dispersal Events of Meconopsis Species

The results above clearly indicate that species from the HDM are positioned near the root of the phylogenetic tree and are distributed across the four major clades, indicating that this region represents the ancestral center of origin for *Meconopsis*. The uplift of the Himalayas occurred much earlier (approximately 20 million years ago), and their topography, shaped by prolonged erosion, formed more continuous ecological corridors that facilitated species migration along altitudinal gradients [32]. The Himalayas exhibit a high rate of species diversification, potentially driven by mountain uplift [33]. Recent evidence from isotope dating and plant fossils suggests that much of the HDM had reached elevations of around 3900 m by the early Oligocene [34,35]; by the early Miocene, the Himalayan region had surpassed 5000 m in elevation [36,37]. The accelerated uplift of the QXP during the middle Miocene coincided with the formation of the HDM, and this orogeny likely contributed to increased chromosome numbers in the eastern Himalayas. The highest chromosomal base diversity in *Meconopsis* species was observed near Mount Everest (Figure S8), which provides further support for the hypothesis that mountain uplift facilitated the evolution of karyotypic diversity, thus contributing to species formation.

By quantifying the dynamics of species formation and dispersal in *Meconopsis*, we showed that the Himalayas have played a key role in the accumulation of species diversity and that climatic fluctuations and dispersal processes have played an important role in shaping alpine plant diversity patterns. These findings support the center of accumulation hypothesis, which posits that species diversity originates in specific geographic centers (e.g., biodiversity hotspots) and subsequently expands through dispersal, contributing to diversity formation at broader spatial scales [38]. Climate instability likely induced habitat fragmentation and resource variability, increasing extinction risks [39]. These conclusions are consistent with global patterns indicating the sensitivity of alpine plants to climate change and underscore the significant role of climatic fluctuations in influencing alpine biodiversity. Furthermore, our findings indicate that future research that integrates genomics, ecological niche modeling, and paleoclimate reconstruction is needed to improve assessments of the effect of specific climatic events on extinction rates and improve the response of species (either through adaptation or migration) to environmental changes. Such efforts will enhance our understanding of the evolutionary history of alpine plant diversity and its resilience or vulnerability to future climate change.

### 3.2. Evolution of Reproductive, Growth, and Fruiting Traits

Examination of the phylogenetic distribution of inflorescence types in *Meconopsis* revealed significant clustering of species sharing the same inflorescence type, flower color, and growth-fruiting strategy on the phylogenetic tree (Figure 2). This pattern suggests that these three traits possess a strong phylogenetic signal within the genus. Ancestral trait reconstruction further indicated that the most likely ancestral state of *Meconopsis* comprised solitary inflorescences with bluish-purple flowers, a combination retained in basal taxa and still common across the genus. These findings support the evolutionary conservatism of reproductive traits in *Meconopsis*, suggesting that solitary, blue-violet flowers may provide adaptive advantages in certain environments. The emergence of racemose inflorescences, particularly in Clades I and II, indicates that shifts in inflorescence architecture have occurred repeatedly during the genus’s evolutionary history. These shifts likely reflect morphological innovations in response to diverse ecological pressures.

Approximately 90% of *Meconopsis* species exhibit one-time fruiting, which represents a dominant evolutionary trend in growth and reproductive strategies. Diversification rate analyses across geographic regions revealed that *Meconopsis* originated in the HDM and subsequently dispersed to other regions, especially the eastern and western Himalayas. This dispersal had profound ecological and evolutionary implications. In the Transantarctic region, species typically exhibit annual one-time fruiting. However, as populations expanded into new environments, some lineages evolved perennial one-time fruiting or even multiple fruiting strategies. These shifts represent adaptive responses to varying ecological pressures. Simultaneously, flower color and inflorescence type underwent transitions, with hues changing from blue-violet to red and yellow, and inflorescence forms evolving from solitary and racemose types to conical or umbel-shaped structures.

These transformations are closely linked to the specific environmental conditions in new habitats, indicating that dispersal requires adaptive modifications for survival in diverse ecological contexts. In particularly harsh environments such as the Himalayas or the QXP, plants often accumulate nutrients over several years to reproduce. This strategy supports both population persistence and ecosystem stability under extreme conditions (e.g., hypoxia, intense ultraviolet radiation, and nutrient-poor soils) and is consistent with the “sky island” effect. The trade-off between vegetative growth and reproduction observed in *Meconopsis* resembles the “r-strategy” often employed by alpine plants [40]. These findings suggest that prolonged nutrient accumulation is essential for survival and successful reproduction in extreme alpine environments. Furthermore, the diversification of reproductive traits in *Meconopsis* is correlated with high net diversification rates across taxa, which underscores the role of reproductive strategies in driving speciation within the genus.

### 3.3. Environmental Interactions and Their Mechanisms

**Figure 7:**
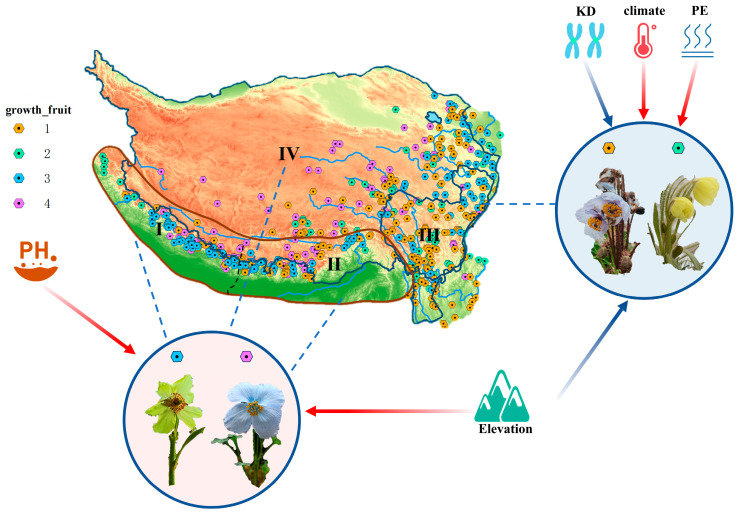
Patterns of environmental factors, karyotypes and growth and fruiting in relation to their effects. Blue arrows represent negative correlations, red arrows represent positive correlations, KD is karyotypic diversity, PE is potential evapotranspiration, The icon in the upper left corner represents plant growth and fruiting (1: one annual, 2: one biennial, 3: one perennial, 4: multiple perennial), and Roman numerals mark the zones of geographic concentration of species in the different clades, and the flowers in the circles are representative plants of *Meconopsis*. The flowers in the circles are representative plant diagrams of the genus *Meconopsis* with different growth and fruiting conditions, with *Meconopsis sulphurea* (perennial single-fruiting) and *Meconopsis bella* (perennial multiple-fruiting) in the bottom circle, and *Meconopsis pseudohorridula* (annual single-fruiting) in the circle on the right. The circles on the right are *Meconopsis pseudohorridula* (one-fruiting annual) and *Meconopsis integrifolia* (one-fruiting biennial).

The multi-year growth cycle of *Meconopsis* plants likely represents an adaptive strategy to the unpredictable and brief growing seasons characteristic of alpine environments. By extending their life cycle, these plants can maximize limited growth opportunities, gradually accumulating the resources required for one-time or even multiple fruiting events. This strategy substantially enhances survival and reproductive success under the resource scarcity and environmental stress typical of alpine ecosystems. The interaction between growth and reproductive strategies is illustrated in Figure 7 Building upon previous studies [41], we extracted relevant ecological factors for more detailed analysis. The karyotypic diversity (chromosome number) of *Meconopsis* was significantly spatially autocorrelated across different distribution areas (Figure S7, Table S4). This pattern was strongly associated with environmental variables such as altitude, mean annual temperature, soil pH, and potential evapotranspiration (Figure 3 and Figure 4). In high-altitude regions, where environmental stress is more pronounced [42], *Meconopsis* plants may increasingly rely on prolonged vegetative growth to accumulate the resources necessary for successful reproduction.

Additionally, rising mean annual temperatures and increased potential evapotranspiration appear to promote the evolution of annual primary fruiting, while neutral to alkaline soil pH favors the evolution of perennial primary or multiple fruiting. Polyploid plants are known to possess competitive advantages, particularly under climate change and environmental stress [43]. Their genomic redundancy and enhanced recombination rates facilitate rapid adaptation, especially in challenging alpine environments [44]. In our study, higher ploidy was associated with deeper flower pigmentation, and darker flowers were more common in perennial species. This may relate to the biosynthesis and regulation of anthocyanins, which are pigments influenced by both genetic pathways and environmental conditions [45]. Polyploid plants generally exhibit darker flower colors due to elevated anthocyanin levels [46]. Species from different clades may undergo dispersal and trait-state transitions across ecological gradients, allowing them to better adapt to diverse niches. These patterns highlight the range of adaptive strategies employed by *Meconopsis* species in alpine habitats.

Polyploid plants typically have extended growth cycles, likely due to their larger size or enhanced metabolic efficiency at the cellular and organ levels, which supports prolonged physiological activity. Polyploidy promotes vegetative growth but may limit the frequency of reproduction, leading to fewer fruiting events. This trade-off may be driven by genetic and physiological barriers that affect reproductive organ development and gametophyte viability, particularly in isolated “sky island” environments where the reproductive capacity and population turnover are reduced. As a result, polyploid *Meconopsis* species must balance longer life cycles with reduced reproductive output to survive under strong selective pressures. Climate and soil jointly shape trait variation, and specific aspects of these environmental variables are particularly influential at the global scale [47]. In this study, we examined climatic, edaphic, and topographic variability among species with different growth and fruiting traits. We further analyzed how environmental factors influenced floral coloration, cladistic and parental lineage relationships, and karyotypic diversity. The spatial distribution of *Meconopsis*, shaped by the “sky island” effect, revealed clear patterns linked to both biotic and abiotic determinants of growth and reproductive traits.

These adaptive strategies reflect how plants balance growth, reproduction, and survival over evolutionary timescales. Karyotypic diversity, reproductive characteristics, ecological conditions, and spatial patterns collectively shape the geographic distributions and adaptive evolution of *Meconopsis*. Elevation facilitates species diversification to a certain extent [48,49]. A comprehensive understanding of these mechanisms will improve our ability to anticipate and mitigate the effects of climate change on alpine ecosystems. Trait variation is also modulated by other factors, including biological interactions (e.g., soil biota), human-induced changes, and localized disturbances such as microclimatic fluctuations [50,51,52,53]. Future research should aim to quantify the effects of microclimatic and anthropogenic disturbances and examine their correlations with global patterns of trait variability. Global changes are accelerating vegetation degradation across many alpine ecosystems, which significantly compromises ecosystem functioning and biodiversity [54]. Therefore, the conservation of alpine biodiversity is an imperative and pressing priority.

## 4. Materials and Methods

### 4.1. Phylogenetic Analysis

In this study, gene sequence data for 47 *Meconopsis* species (As documented in the Flora of China (FOC), 87.04% species diversity of the Genus) were obtained from the NCBI GenBank database [55], along with sequences from 12 outgroup species. The genomic length of each species measures 4075 base pairs. Due to ongoing debate on the fossil record of the Papaveraceae family [56,57,58,59], we followed the recommendations of previous studies [60] and incorporated fossil calibrations from related families to construct a more robust temporal phylogeny. This yielded a dataset of 82 species, which was used to build a more reliable phylogenetic tree. The chloroplast gene regions selected for analysis included *trnL-trnF*, *matK*, *rbcL*, and *ndhF*. A preliminary phylogenetic tree was generated for each individual gene marker, followed by concatenation of the sequences (gene counts per marker are listed in Table S5).

Sequence alignment was performed using MAFFT v7.023 with the G-INS-i algorithm [61], and the alignments were subsequently trimmed using default settings in Gblocks. Initial maximum likelihood (ML) trees were constructed for each marker gene, and one representative gene sequence per species was selected to ensure accuracy in the merging process. The four marker genes were then concatenated using ACOPTools_v2 and 1000 bootstrap replicates; the best-fitting nucleotide substitution model was determined via ModelFinder. Phylogenetic inference was carried out using both maximum likelihood and Bayesian methods. ML trees were inferred using IQ-TREE [62], and Bayesian inference (BI) was performed using MrBayes 3.2.7 [63,64].

Due to the lack of a reliable fossil record for Papaveraceae, divergence times were estimated using previously proposed secondary calibration points [60]. Outgroup taxa from the Ranunculaceae, Berberidaceae, Menispermaceae, and Lardizabalaceae families were used to calibrate divergence times within the *Meconopsis* phylogeny.

### 4.2. Estimation of Divergence Times

Divergence times were estimated using secondary calibration points for *Meconopsis* species and outgroup taxa, following previous studies [60]. The crown age of the Papaveraceae was estimated at 120.77 Ma (95% highest posterior density [HPD]: 117.91–123.77 Ma). For the Ranunculaceae, fossil-based crown ages ranging from 70.8 to 88.8 Ma (95% HPD) [65] were used for node calibration and temporal scaling in BEAST v2.3.0 [66,67,68]. Normal prior distributions were assigned to all calibration points, with 95% confidence intervals. An uncorrelated relaxed log-normal clock was applied under a birth–death speciation model, and the GTR model was used for nucleotide substitution. The Gamma Category Count was set to 4, and additional parameters, including substitution rate and shape, were estimated during the analysis.

Independent Markov Chain Monte Carlo (MCMC) runs were executed using a chain length of 100,000,000 iterations, sampling every 10,000 generations. Convergence and effective sample sizes were assessed using Tracer v1.4 (http://tree.bio.ed.ac.uk/software/tracer/) (accessed on 3 November 2024) to ensure that all parameters reached stationarity [69]. Divergence times and corresponding 95% HPD intervals for each species were summarized using TreeAnnotator v1.10.4 [70], and the resulting time-calibrated phylogenetic tree was visualized using FigTree v1.4.4 (http://tree.bio.ed.ac.uk/software/figtree/) (accessed on 7 October 2024).

### 4.3. Ancestral State Reconstructions

Ancestral region reconstruction was conducted by thoroughly analyzing distribution point data from *Meconopsis* plant specimens. Based on these data, the distribution of *Meconopsis* was divided into four primary regions: the HDM, the plateau region of the QXP, the eastern Himalayas, and the western Himalayas. Model testing was performed using RASP v3.2, and the model with the highest AICc_wt value was selected. The BAYAREALIKE+J model was ultimately chosen for ancestral region reconstruction [71,72].

We also mapped four key traits of *Meconopsis*: inflorescence type, flower color, life history (annual vs. perennial), and fruiting habit (single vs. multiple fruiting). Trait data were compiled from multiple sources, including the *Flora of China* (https://www.iplant.cn/foc/), *eFlora of India* (https://efloraofindia.com/), and relevant literature [73,74]. For missing or unavailable data, we supplemented trait information through field observations of live plants and herbarium specimens [75]. These observations were further refined by expert evaluations of root morphology, habitat conditions, and a comprehensive literature review [76,77,78].

All four traits, which were discrete (Table S6), were systematically coded for mapping: inflorescence type, flower color, life history (annual/perennial), and fruiting habit (single or multiple fruiting) (https://itol.embl.de/). By integrating the ancestral region reconstruction with trait mapping, we aimed to elucidate the geographic distribution patterns of *Meconopsis*, as well as the evolutionary dynamics underlying its growth forms, reproductive traits, and fruiting strategies.

### 4.4. Analysis of Interactions Between Adaptive Traits and Ecological Factors

ArcGIS v10.4 was used to process large-scale raster data, including climate layers (e.g., temperature and precipitation), to extract environmental information for specific regions [79]. In this study, we first obtained latitude and longitude records for *Meconopsis* species from the GBIF database (https://www.gbif.org/, accessed on 4 December 2024) [80] using the R 4.3.0 packages rgbif and CoordinateCleaner [81]. The data were cleaned by removing duplicate entries and outliers [82]. Subsequently, 19 bioclimatic variables (from https://worldclim.org/) (accessed on 19 November 2024), the HWSD soil dataset (https://gaez.fao.org/pages/hwsd) (accessed on 29 November 2024), elevation data, potential evapotranspiration [83], and the aridity index [84] were compiled, resulting in a total of 41 environmental variables (excluding latitude and longitude). These variables cover a wide range of aspects such as climate, soil, topography and water balance, which can reflect the influence of the environment on biological reproduction traits in a more comprehensive manner. By comprehensively analyzing these variables, the complex mechanism of environmental factors on biological reproduction can be more deeply understood. For the aridity index, missing values were imputed with the median because the data layer covered only the QXP region, and some distribution points fell outside its bounds. Chromosome data for each species were retrieved from the Chromosome Count Database (https://ccdb.tau.ac.il). Environmental variable values were extracted for each set of geographic coordinates using ArcGIS [85]. These data, combined with the trait and karyotype data, yielded a total of 52 variables (Table S3; see Supplementary File “total factors.csv” for complete data). Assuming growth and fruiting patterns were consistent, the remaining 49 variables (excluding latitude and longitude) were grouped into three categories. Heatmap analyses were conducted separately for each group, and the 18 most highly correlated variables were selected for principal component analysis (PCA). From these, 10 variables with the strongest correlations were selected for mixed-effects modeling. 

Before statistical analysis, outliers (e.g., extreme elevation values or missing data) were removed from the 18 selected variables, and the data were standardized to a mean of 0 and a standard deviation of 1. Multilevel regression analysis was employed to account for nested data structures and variable interactions across hierarchical levels. Linear mixed-effects models [86], implemented using the R packages lme4 and tidyverse, were applied to assess the influence of karyotypic traits (e.g., chromosome number, ploidy level, and flower color variation) and environmental factors on the growth and fruiting of alpine species. Given the common issue of spatial dependence in ecological data, particularly spatial autocorrelation, we followed methodologies from prior studies [87] and employed ordinary least squares (OLS), spatial lag models (SLMs), and spatial error models (SEMs) using the spdep (1.3.6) and sf (1.0.16) packages. Additionally, the vegan and dplyr packages were used to test linearity assumptions, while non-metric multidimensional scaling, along with box-and-line plots, was employed to evaluate the effects of ecological variables on the growth, fruiting, and karyotypic characteristics of *Meconopsis* [88].

### 4.5. Diversification Rate Estimation

The GeoSSE model, a maximum likelihood framework for analyzing diversification rates, was used to investigate the geographic distributions of species and their evolutionary dynamics across different environments [89]. To specifically address the geographic sampling, we selected 47 *Meconopsis* species for the analysis. The distribution range of each species was codified into discrete states: species endemic to the Hengduan Mountains were coded as state 1, those restricted to the Himalayas were coded as state 2, and widespread species occurring in both regions were coded as state 0. A time-calibrated phylogenetic tree was pre-processed using the ape package in R [90], with redundant branches between ingroup species and outgroups removed to ensure analytical accuracy. To account for phylogenetic uncertainty, 100 phylogenetic trees were randomly selected from the BEAST output. Finally, the GeoSSE model was implemented using the diversitree package in R. Each tree was analyzed using 1000 MCMC generations to estimate diversification rates and evaluate the influence of ecological environments, geographic regions, and adaptive strategies on species diversification.

The MuSSE model has the advantage of multi-state analytical capability, flexibility in parameter estimation, diversity of research hypotheses, adaptability to complex evolutionary processes, and provision of more accurate estimates in the study of trait diversification rates. To assess diversification rates associated with specific traits, we conducted a Multistate Speciation and Extinction (MuSSE) analysis [89]. Speciation rates (λ), extinction rates (μ), and transition rates between trait states (q) were estimated using a maximum likelihood framework. Eight competing models were fitted to the data, and the best-fitting model was selected based on model comparison criteria. Following this, Bayesian MCMC analyses were performed to estimate posterior distributions for the model parameters, which allowed the estimation of speciation and extinction rates for each trait state. Net diversification rates were calculated by subtracting extinction rates from speciation rates.

## 5. Conclusions

Biotic and abiotic factors are central to the mechanisms driving the formation and maintenance of alpine plant diversity. Our integrative analysis demonstrates that the geographic distribution and adaptive evolution of the genus *Meconopsis* are jointly shaped by intrinsic factors—specifically chromosomal diversity and reproductive traits—and extrinsic ecological conditions and spatial patterns. We revealed that *Meconopsis* originated in the HDM, and that tectonic uplift in the eastern and western Himalayas, characterized by pronounced topographic heterogeneity and climatic fluctuations, significantly promoted both chromosomal base diversification and speciation. Furthermore, our findings established the *Meconopsis* geographic pattern under the influence of the “sky island” effect for the first time, indicating that ecological heterogeneity, particularly in climate and soil, collectively shapes its distribution. The results also highlight the unidirectional influence of polyploidy on floral coloration, growth, and fruiting patterns, potentially owing to the physiological and ecological advantages that polyploidy confers in high-altitude environments. This integrated biogeographic and ecological framework offers valuable aid in understanding the growth–fruiting strategies and reproductive adaptations of alpine plants.

## Data Availability

Climate data used in the study can be obtained from the global climate database WorldClim (https://worldclim.org/), soil data can be obtained from HWSD (Harmonized World Soil Database, HWSD) (http://www.fao.org), potential evapotranspiration development dataset, and aridity index are available from the National QXP Data Center (https://data.tpdc.ac.cn/home), and chromosome data are available from the CCDB (https://ccdb.tau.ac.il). Sequence numbers of the genes in this study are available from NCBI (https://www.ncbi.nlm.nih.gov/).

## Supplementary Materials

The following supporting information can be downloaded at: https://www.mdpi.com/article/10.3390/plants14243741/s1, Figure S1: ML tree (IQ-TREE) constructed based on chloroplast marker genes.; Figure S2: BI tree (MrBayes) constructed based on chloroplast marker genes; Figure S3: Plot of temporal dispersion estimates using BEAST; Figure S4: Heatmap for each of the 49 factor categories, selecting the 10 variables with higher correlations; Figure S5: Interaction between two variables under linear mixed model; Figure S6: Different colored dots represent the status of *Meconopsis* under different basal changes, and the line direction represents the change trend; Figure S7: Spatial autocorrelation was used to analyze the effect of karyotypic diversity (chromosome number) on growth and fruiting; Figure S8: Geographic distribution of *Meconopsis* chromosome bases; Figure S9: Plot of PCA cumulative variance and MantelTest goodness-of-fit assessment in factor dimensionality reduction; Figure S10: PCoA with NMDS analysis. Table S1: Reconstruction of ancestral regions model detection results; Table S2: The latitudinal and longitudinal data of *Meconopsis* and related genera were used to classify the regions into which the species were concentrated; Table S3: The latitude and longitude data of *Meconopsis* were extracted from GBIF; Table S4: Spatial autocorrelation analysis; Table S5: Gene sequence numbers for *Meconopsis* and outgroups; Table S6: Characterization data for *Meconopsis*.
