# Supplementary material for: Adaptive Strategies Mediating the Diversification of Alpine Plants: The Case of the Himalayan Blue Poppy (Meconopsis, Papaveraceae)
Source: Plants (Basel). 2025 Dec 8;14(24):3741. doi: 10.3390/plants14243741 (PMC12736773; doi:10.3390/plants14243741)
Supplement: Supplementary file 1 [file plants-14-03741-s001.zip › plants-3932368-supplementary/Supplementary File/appendix.pdf]

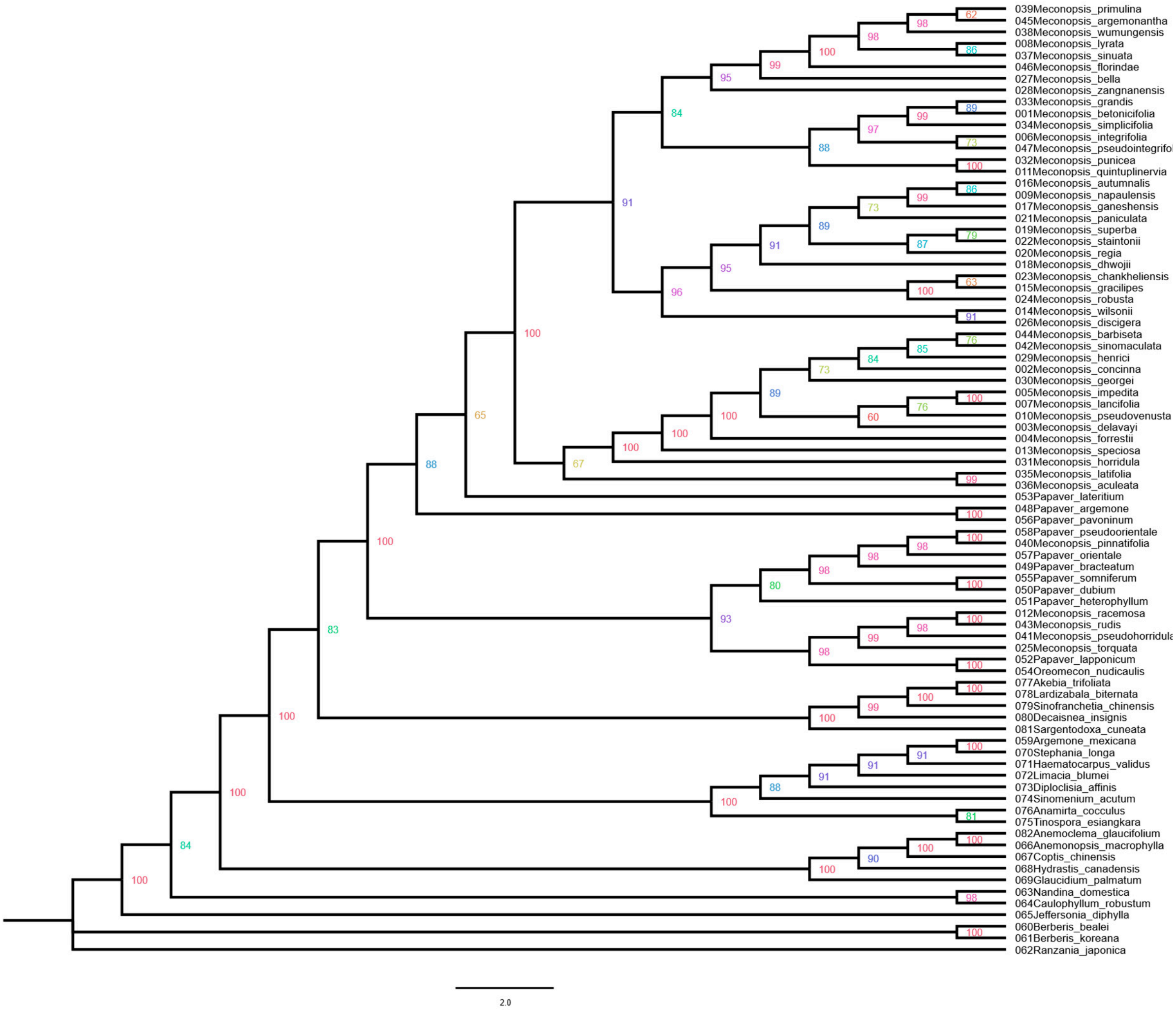

Figure S1. ML tree (IQ-TREE) constructed based on chloroplast marker genes. Numbers represent support, and different line lengths represent different genetic distances.

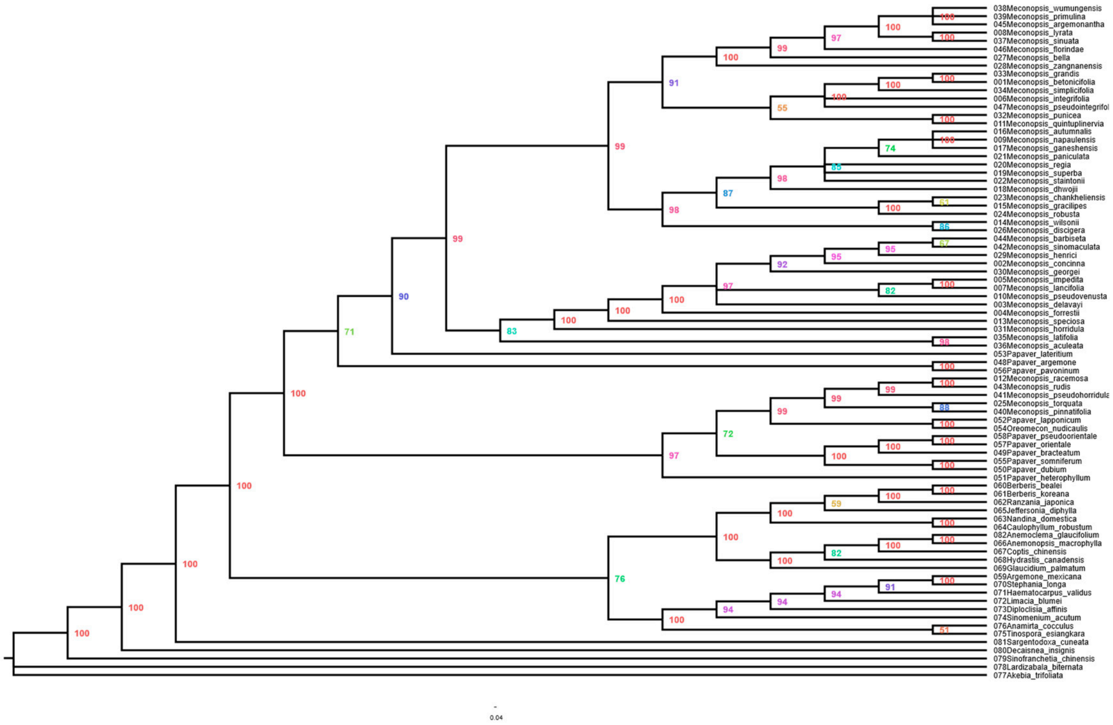

Figure S2. BI tree (MrBayes) constructed based on chloroplast marker genes. Numbers represent support, and different line lengths represent different genetic distances.

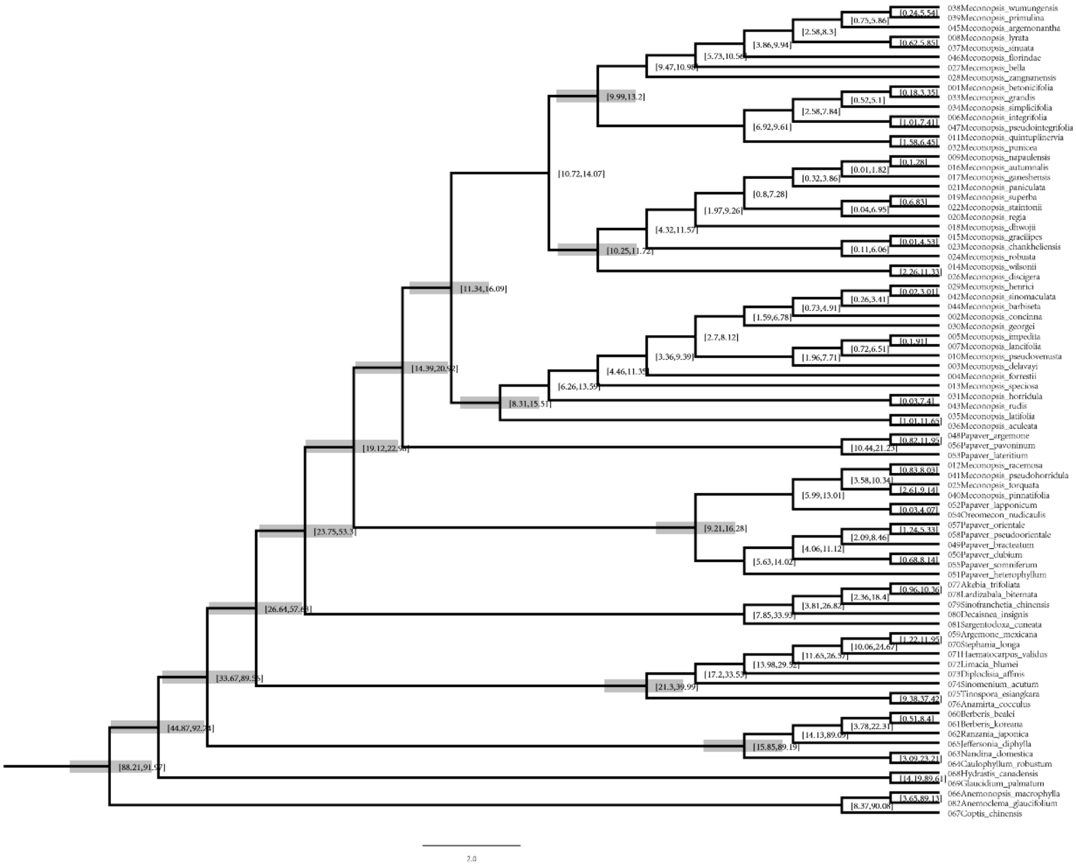

Figure S3. Plot of temporal dispersion estimates using BEAST, numbers represent estimated million-year ranges, and the range of numbers in the figure represents the approximate time to 95% maximum a posteriori density.

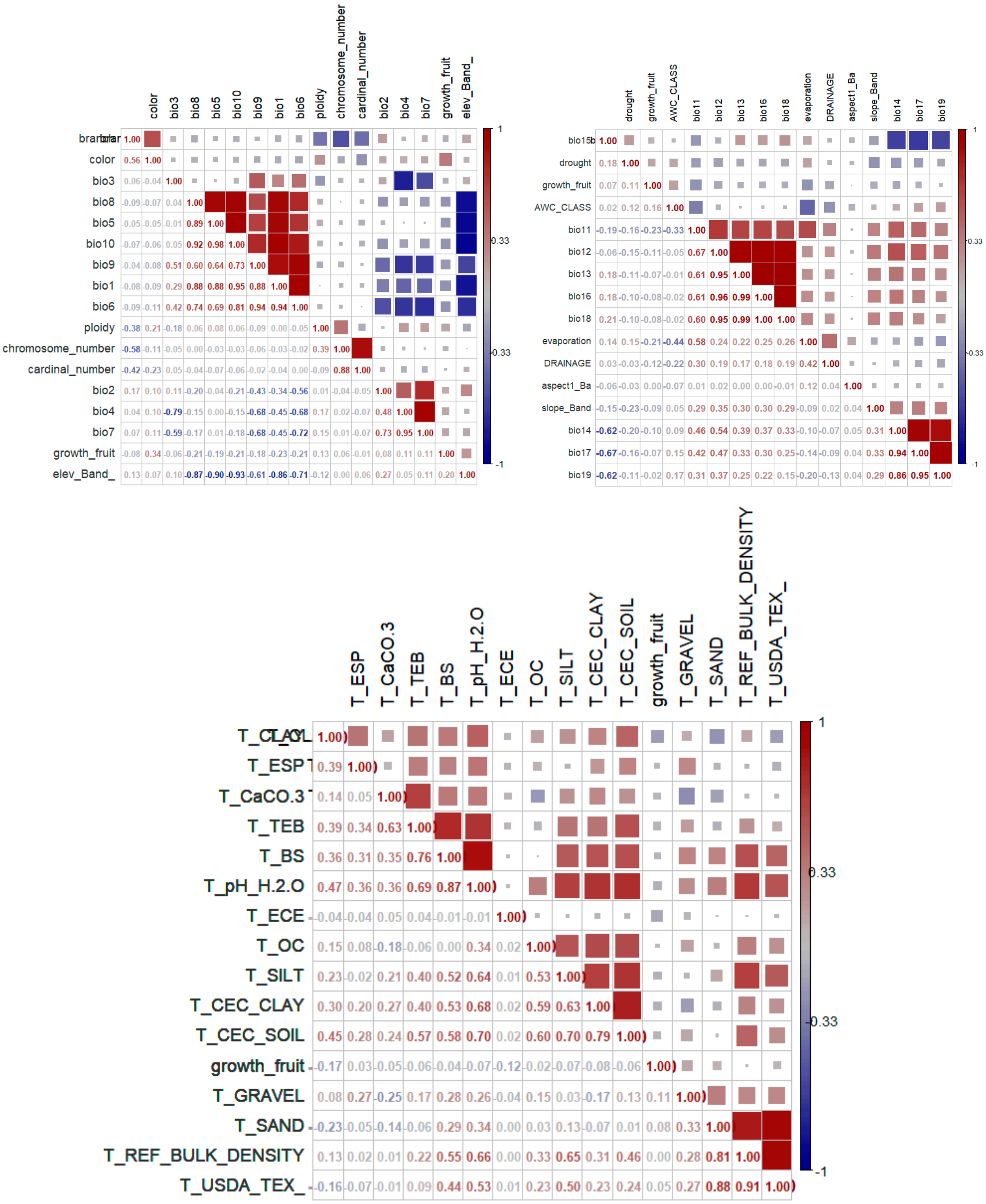

Figure S4. Heatmap for each of the 49 factor categories, selecting the 10 variables with higher correlations. The text of the horizontal and vertical coordinates is the name of the variable, the number represents the correlation coefficient, blue is a negative correlation, brown-red is a positive correlation, the darker the color, the stronger the correlation, the square represents the significance, the smaller the square, the less significant, and vice versa. The meanings of the horizontal and vertical coordinates are shown in Table C.1.

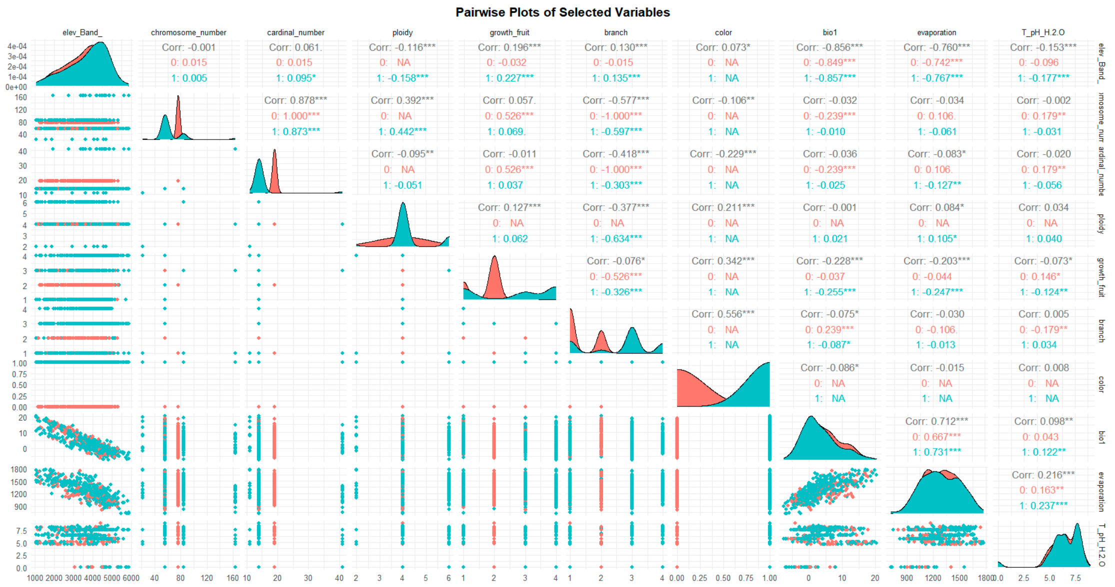

Figure S5. Interaction between two variables under linear mixed model. Variables with high correlation were filtered by heat map (Fig. D.1), and the relationship between the two variables was plotted using Ggally package classified by flower color shade. Cyan represents dark flowers, orange represents light flowers, dots represent the status of species distribution points, curves represent changes in variables, the number of * represents significance, and numbers represent correlation coefficients.

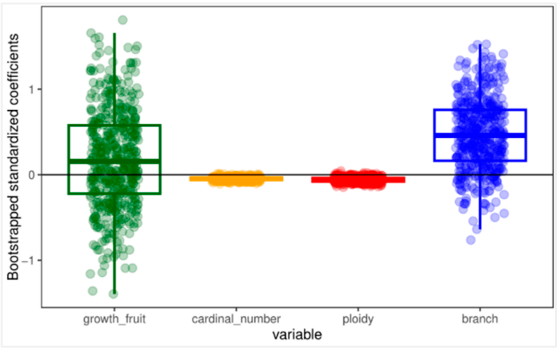

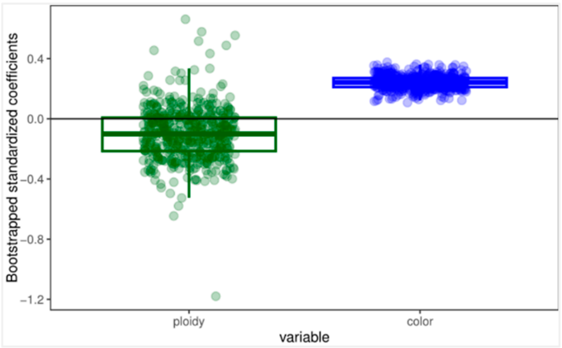

Figure S6. Different colored dots represent the status of *Meconopsis* under different basal changes, and the line direction represents the change trend. The bootstrapped standardized coefficients of the fixed terms were obtained from the mixed-effects model in Figures c and d. Figure c explores the effects of the four variables on flower color. Figure d explores the effect of biennial variables on growth and fruiting. Numbers are coefficient values, with positive and negative representing positive or negative effects. See Table C.1 for specific meanings of horizontal and vertical coordinates.

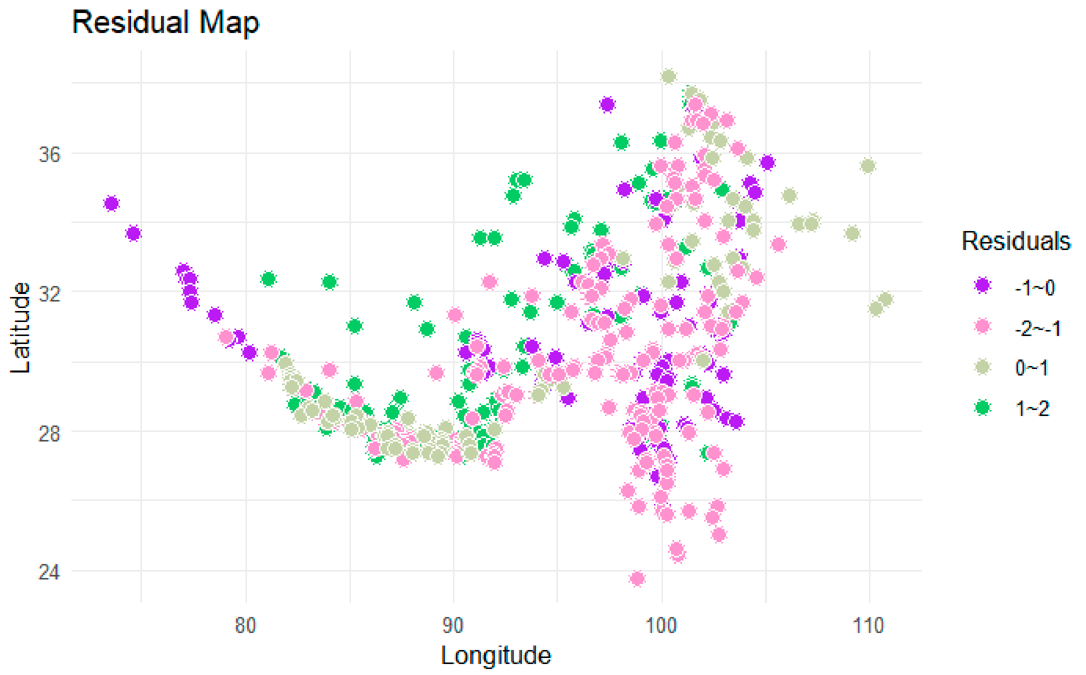

Figure S7. Spatial autocorrelation was used to analyze the effect of karyotypic diversity (chromosome number) on growth and fruiting. Horizontal coordinates represent longitude, vertical coordinates represent latitude, numerical intervals on the right represent the range of residual values, different colors represent different residual intervals, and dots represent species-specific geographic locations.

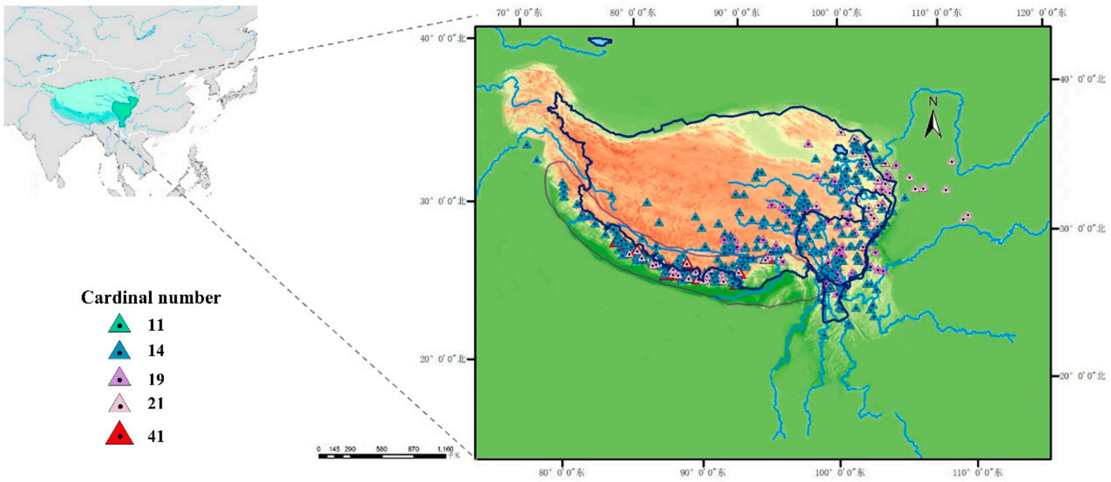

Figure S8. Geographic distribution of *Meconopsis* chromosome bases. Different colored triangles represent different chromosome bases, and the numbers around the positive boxes represent latitude and longitude, with the Tibetan Plateau, the Hengduan Mountains, and the Himalayas mainly shown in the figure.

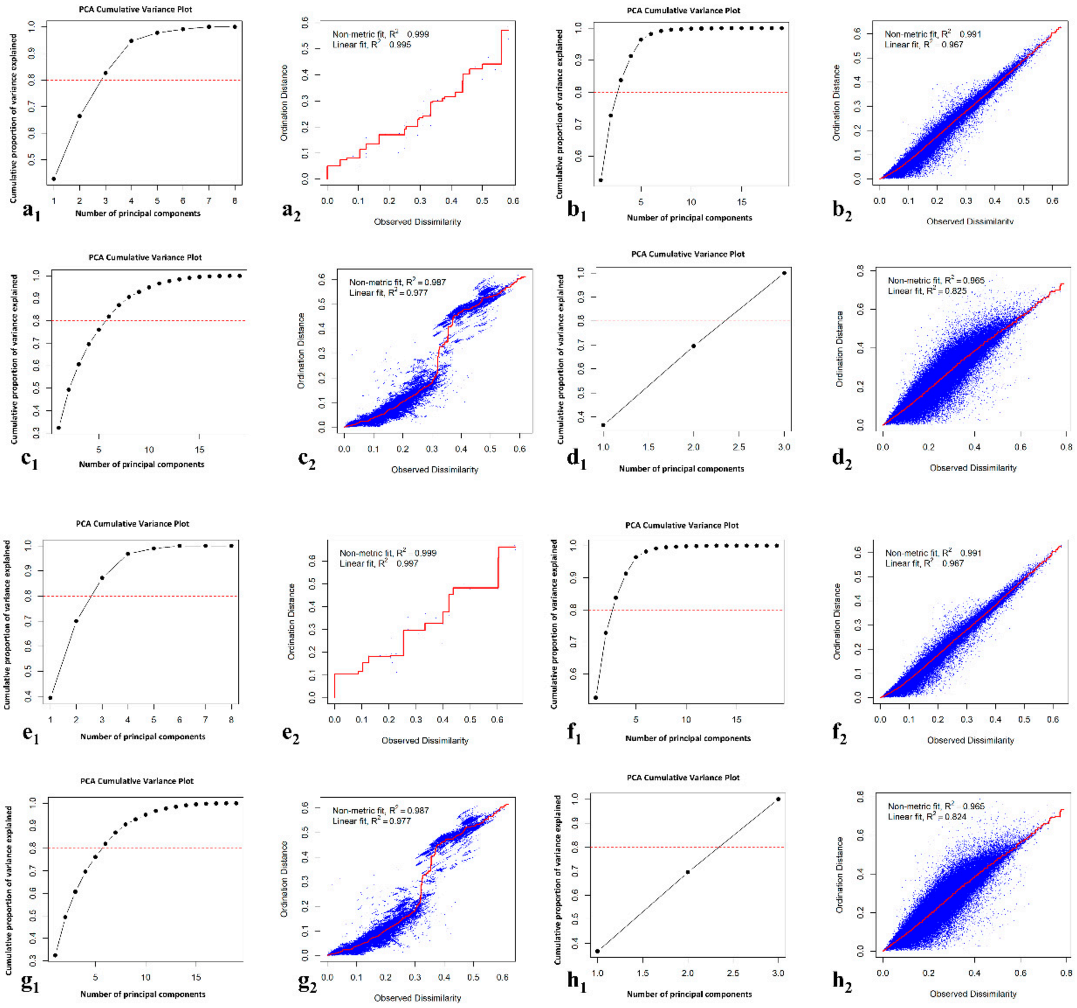

Figure S9. Plot of PCA cumulative variance and MantelTest goodness-of-fit assessment in factor dimensionality reduction. Classification by growth and fruiting, a_1-a_2 :contains 8 biological factors; b_1-b_2 : contains 19 climate factors; c_1-c_2: contains 19 soil factors; d_1-d_2: contains 3 geographic factors. Taking the four main branches of the classification, e_1-e_2: contains 8 biological factors; f_1-f_2: contains 19 climate factors; g_1-g_2: contains 19 soil factors; h_1-h_2: contains 3 geographic factors. The left plot represents the number of principal components in the horizontal scale and the proportion of cumulative explained variance in the vertical scale. The right-hand plots for each group are the fit plots for the annual amount of individual non-metric perennial dimensional scaling analyses (NMDS) for assessing the quality of the NMDS ordination as well as the fit between the data and the NMDS ordination results. The R^2 approximation of the non-metric fit is close to 1, indicating that the sorting results reflect the structure of the original data well, and the R^2 of the linear fit indicates a good fit to the NMDS results.

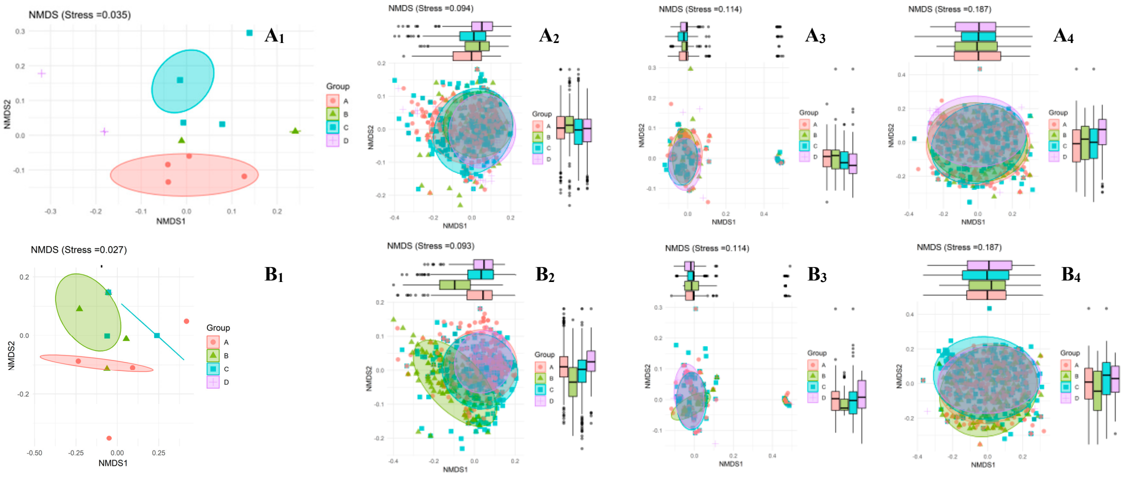

Figure S10. PCoA with NMDS analysis. The 49 classes of data were divided and then downscaled, the horizontal and vertical coordinates in figure represent the two main multidimensional scales, the ellipses represent the 95% confidence intervals, the Groups in A1-A4 represent the growth and fruiting conditions (A: one annual fruiting, B: one biennial fruiting, C: one perennial fruiting, and D: many perennial fruiting), and the Groups in B1-B4 represent the clade categorization (A. Clade I, B:Clade II, C: Clade III, D: Clade IV), and each point represents a sample.A1 and B1 are downscaled to 8 biological factors, A2 and B2 are downscaled to 19 climatic factors, A3 and B3 are downscaled to 19 soil factors, and A4 and B4 are downscaled to 3 topographic factors. The position of the samples in the plot reflects their similarity in the original high-dimensional space, and the box-and-line plots at the edges represent the differences in the distribution of the corresponding different groups on the NMDS.
